# A prospective cohort study examining the association of claw anatomy and sole temperature with the development of claw horn disruption lesions in dairy cattle

**DOI:** 10.3168/jds.2023-23965

**Published:** 2024-04

**Authors:** Bethany E. Griffiths, Matthew Barden, Alkiviadis Anagnostopoulos, Cherry Bedford, Helen Higgins, Androniki Psifidi, Georgios Banos, Georgios Oikonomou

**Affiliations:** 1 Department of Livestock and One Health, Institute of Infection, Veterinary and Ecological Sciences, University of Liverpool, Leahurst Campus, Liverpool, CH64 7TE, United Kingdom; 2 Department of Clinical Science and Services, Royal Veterinary College, North Mymms, Hertfordshire, AL9 7TA, United Kingdom; 3 Animal and Veterinary Sciences, SRUC, Roslin Institute Building, Easter Bush, Midlothian, EH25 9RG, United Kingdom

**Keywords:** lameness, digital cushion, sole temperature, sole thickness

## Abstract

Foot characteristics have been linked to the development of sole lesions (sole hemorrhage and sole ulcers) and white line lesions, also known as claw horn disruption lesions (CHDL). The objective of this study was to examine the association of claw anatomy and sole temperature with the development of CHDL. A cohort of 2,352 cows was prospectively enrolled from 4 UK farms and assessed at 3 time points: before calving (T1-precalving), immediately after calving (T2-calving), and in early lactation. At each time point body condition score was recorded, a thermography image of each foot was taken for sole temperature measurement, the presence of CHDL was assessed by veterinary surgeons, and an ultrasound image was taken to retrospectively measure the digital cushion and sole horn thickness. Additionally, at the postcalving time point, foot angle and heel depth were recorded. Four multivariable logistic regression models were fit to separately examine the relationship of precalving and postcalving explanatory variables with the development of either white line lesions or sole lesions. Explanatory variables tested included digital cushion thickness, sole horn thickness, sole temperature, foot angle, and heel depth. Farm, parity, body condition score, and presence of lesion at the time of measurement were also included in the models. A thicker digital cushion shortly after calving was associated with decreased odds of cows developing sole lesions during early lactation (odds ratio [OR]: 0.74, 95% confidence interval [CI]: 0.65–0.84). No association was found between digital cushion thickness and development of white line lesions. Sole temperature after calving was associated with increased odds of the development of sole lesions (OR: 1.03, 95% CI: 1.02–1.05), and sole temperature before and after calving was associated with the development of white line lesions (T1-precalving OR: 1.04, 95% CI: 1.01–1.07; T2-calving OR: 0.96, 95% CI: 0.93–0.99). Neither foot angle nor heel depth was associated with the development of either lesion type. However, an increased sole horn thickness after calving reduced the odds of cows developing sole lesions during early lactation (OR: 0.88, 95% CI: 0.83–0.93), highlighting the importance of maintaining adequate sole horn when foot trimming. Before calving, animals with a lesion at the time of measurement and a thicker sole were more likely to develop a sole lesion (OR: 1.23, 95% CI: 1.09–1.40), compared with those without a sole lesion. The results presented here suggest that white line and sole lesions may have differing etiopathogenesis. Results also confirm the association between the thickness of the digital cushion and the development of sole lesions, highlight the association between sole horn thickness and sole lesions, and challenge the potential importance of foot angle and heel depth in the development of CHDL.

## INTRODUCTION

Lameness in dairy cattle is highly prevalent and associated with a range of negative effects (Espejo et al., 2006; Griffiths et al., 2018; Bran et al., 2019). Lameness is not only a serious welfare concern but also a production and economic concern to the industry (Charfeddine and Pérez-Cabal, 2017; Whay and Shearer, 2017; Omontese et al., 2020). Lameness is a clinical symptom of underlying disease, with pathology most often occurring within the foot (Murray et al., 1996).

Sole ulcers and sole hemorrhage (sole lesions) are the results of inflammation and derangement of horn production resulting from an insult applied to keratinocytes found within the corium (Bergsten, 1994). If severe, horn production will cease, and the resulting deficit exposes sensitive tissues to the environment (sole ulcer). Sole hemorrhage is thought to represent a different stage or severity of insult applied (Bergsten, 1994; Lischer and Ossent, 2001; Newsome et al., 2016). White line lesions are thought to arise from a similar process to sole ulcers and sole hemorrhage (Lischer and Ossent, 2001). The environmental risk factors associated with white line lesions are, however, different than those for sole hemorrhage and sole ulcers (Barker et al., 2009), and white line lesions are located at the junction between the sole and the hoof wall rather than the sole. Claw horn disruption lesion (**CHDL**) is the collective term used for sole ulcers, sole hemorrhage, and white line disease, with the term describing the presentation rather than any underlying etiopathogenesis (Hoblet and Weiss, 2001). These lesions, particularly sole ulcers and white line disease, are the main causes of lameness in dairy cattle and are most prevalent during the early lactation period (Hoblet and Weiss, 2001; Bicalho et al., 2009).

Compressive forces that damage the corium during normal weight bearing and foot strikes have been hypothesized to be the insult behind the development of CHDL (Räber et al., 2004). The suspensory apparatus of the cow is limited, and great reliance is placed upon the supportive apparatus, which includes the digital cushion (Räber et al., 2004, 2006). The digital cushion, located between the distal phalanx and the corium, comprises 3 soft tissue pads, composed of fat and connective tissue (Räber et al., 2004, 2006). These are hypothesized to protect the corium from the compressive forces placed upon it during weight bearing and ambulation. The size of this cushion has been associated with CHDL in several cross-sectional and longitudinal studies, with thinner digital cushions being associated with the development of CHDL (Bicalho et al., 2009; Machado et al., 2011; Newsome et al., 2017a,b).

When the thickness of the sole horn is inadequate, it is hypothesized that the load-bearing capacity of the claw is disrupted, triggering compression of the corium (Archer et al., 2015). Sole horn that is too thin does not provide adequate protection from external mechanical and chemical insults (Manske, 2002; van Amstel et al., 2004). Overgrown soles are associated with displaced loading forces through the hoof, altered toe angles, and an increased risk of lameness-causing lesions (Manske et al., 2002; van der Tol et al., 2004). Although the importance of appropriate load bearing is well known, there is a paucity of peer-reviewed evidence directly linking sole horn thickness to CHDL.

Appropriate load bearing is intrinsically linked to both foot angle and heel depth. The evidence for the importance of foot angle and heel depth in CHDL development is mainly driven by studies examining them as breeding traits (Boelling and Pollott, 1998; Oikonomou et al., 2013). An increase in foot angle was associated with a reduction in the likelihood of clinical lameness; however, feet were not examined for specific lesions such as CHDL (Wells et al., 1993).

Inflammation is thought to play a key role in the development of CHDL (Wilson et al., 2022), being both a sequela and a possible inciting insult (Watson et al., 2022). The temperature of the extremities and skin is largely dependent upon underlying blood circulation and the rate of tissue metabolism (Berry et al., 2003). An insult, which could be biomechanical or inflammatory in nature, causing damage to keratinocytes within the corium would affect blood circulation. This resulting temperature change would alter the heat pattern, allowing infrared thermography to be a potentially useful tool for detecting elevated temperatures associated with foot lesions in dairy cattle (Stokes et al., 2012; Alsaaod et al., 2015). Sole temperature is also associated with increased locomotion scores (Oikonomou et al., 2014). Sole temperature therefore may be a proxy for determining inflammation within the claw at key time points.

Parturition and the management and biological changes that occur in preparation for the next lactation within the transition period are critically important to the health and production of dairy cattle (Drackley, 1999). Historically the etiopathogenesis of CHDL has not been associated with parturition; however, prompted by key research undertaken by Tarlton et al. (2002), who showed increased laxity in supportive structures within the foot at calving, it is now regarded as an area of increasing interest.

In summary, the development of CHDL is multifactorial, representing the interrelationship of genetic (Barden et al., 2022; Li et al., 2023), anatomic, management (Griffiths et al., 2018), and environmental components (Barker et al., 2009; Rutherford et al., 2009; Solano et al., 2015). The objective of the present study was to assess claw anatomy and sole temperature and their association with CHDL by examining key measurements during the transition period and the subsequent development of sole and white line lesions during early lactation in a large cohort of well-monitored UK dairy cows. The null hypothesis was that claw anatomy and sole temperature have no association with the development of sole or white line lesions.

## MATERIALS AND METHODS

This study was conducted under the ethical approval of the University of Liverpool Research Ethics Committee (VREC269a, VREC466ab).

### Study Design

This prospective cohort study was designed to evaluate the study population at 3 key time points during a lactation cycle. Animals were enrolled from 4 UK commercial dairy farms (farms **A–D**) in the Northwest of England and North Wales. Due to the practicalities of frequent visits, the farms were selected for convenience based on proximity and their willingness to participate. A total of 2,352 animals registered as Holstein and expected to calve between April and December 2019 were prospectively enrolled, with no further inclusion or exclusion criteria employed. Data were collected weekly or twice weekly by the researchers on each farm from February 2019 to March 2020. Animals were examined at 3 time points (Supplemental Figure S1, https://data.mendeley.com/datasets/vk42vz8cht/1; Griffiths, 2023): before parturition (**T1-precalving**), immediately after parturition (**T2-calving**), and in early lactation (**T3-early**). Animals were enrolled for 6 mo, after which additional enrollments ceased due to resource constraints and the practicalities of sampling at multiple time points.

### Study Population

All farms calved cows all year-round. Farms A, B, and C housed lactating cattle year-round. Farm D housed high-yielding cattle year-round, with low-yielding animals grazed during summer. All farms housed their lactating cattle on freestall cubicles with deep sand bedding (farms B and C), mattresses with a layer of sand (farm D), or mattresses with a layer of sawdust (farm A). All herds had rubber matting in the parlor and grooved concrete in the passageways, loafing areas, and collecting yards. Cows were milked thrice daily on farms A, B, and C, with recorded 305-d milk yields of approximately 11,000 to 11,500 L. Farm D milked twice a day, recording a 305-d milk yield of approximately 9,000 L. Parous cows in all herds were prophylactically foot trimmed twice a year at drying off and approximately 60 to 100 d after parturition. All herds regularly foot-bathed lactating cows after milking. Farm A foot-bathed 3 times a week with either copper sulfate or formalin; farm B foot-bathed twice daily with formalin; farm C foot-bathed cows daily with either copper sulfate or formalin; and farm D foot-bathed cows 3 times a week with formalin.

### Data Collection

Digital cushion thickness, sole horn thickness, foot angle, and heel depth, in addition to sole temperature, were measured to examine the association between claw anatomy traits and sole temperature, and the development of CHDL. Measurements were taken at T1-precalving and T2-calving, unless otherwise stated. The presence of CHDL was identified at all time points (T1-precalving: −90 to −14 d relative to parturition; T2-calving: 0 to +14 d; T3-early: +40 to +130 d), whereas foot angle and heel depth were recorded only at T2-calving. Data were recorded using Microsoft Access 2010 (Microsoft Corp.).

#### Lesion Identification

All claws from multiparous animals were prophylactically trimmed at T1-precalving and T3-early, according to a modified Dutch 5-step method (Toussaint-Raven, 1989), which included deeper and wider modeling of the lateral claw of hind feet compared with the traditional method. At T2-calving, feet were not prophylactically trimmed; however, a thin layer of horn was removed to reveal the presence of any sole or white line lesions. Heifers were prophylactically trimmed in the same manner at T3-early and T2-calving as their multiparous herd mates; however, no prophylactic trim occurred at T1-precalving. Instead, a thin layer of horn was removed as described at T2-calving. Lesions were then recorded according to the International Committee for Animal Recording claw health atlas (Egger-Danner et al., 2014) and then graded. Supplemental Table S1 (https://data.mendeley.com/datasets/vk42vz8cht/1; Griffiths, 2023) describes the grading system for sole lesions and white line lesions; however, broadly, these scores are comparable to absent (score 0), mild (score 1), moderate (score 2), and severe (score 3). Over 90% of lesion assessments were performed by a single researcher, and the remainder were done by a further 3 researchers. All researchers who undertook the scoring of lesions were qualified veterinary surgeons. On farm C, only hind feet were examined for the presence of lesions at T2-calving, to reduce handling time due to a large number of recently calved cows. Lesions were treated according to standard practice: loose horn was trimmed, and the non-affected claw was blocked (Shearer and van Amstel, 2003).

#### Sole Temperature

Animals were restrained in a foot trimming crush, and each hind foot was lifted. If required, gross contamination of the foot was wiped with paper towel. A thermography image was taken of the plantar aspect of each hind foot at a 30-cm approximate distance. The thermography image was taken using a FLIR E8-XT (FLIR Systems) camera with an emissivity value set at 0.95.

#### Digital Cushion Thickness and Sole Horn Thickness

An ultrasound image was taken of the hind left lateral claw once the leg was lifted, after lesion identification, including the removal of horn, had taken place. Time restraints meant that not all claws could be examined by ultrasound. Due to the increased frequency of CHDL, the hind lateral claw was chosen (Murray et al., 1996), with the lateral hind left claw arbitrarily chosen over the lateral hind right claw. The claw was examined using B-mode ultrasonography as described by Kofler et al. (1999). A 5-cm linear probe inside a gel standoff was used with a Dramiński Vet 4 Mini ultrasound machine (Dramiński S. A.). The frequency was set to 6 MHz, and an image depth of 4 cm was used. The probe was placed on the midline of the sole, and the image was stored once the digital cushion, distal phalanx, tuberculum flexorum, and interface of the sole horn and sole soft tissues were observed.

#### Foot Angle and Heel Depth

At T2-calving, foot angle and heel depth of each hind lateral claw were measured after the leg had been lifted but before any trimming. Heel depth was measured using a ruler accurate to 1 mm, from the coronary band where the perioplic horn becomes hard vertically to the plantar aspect of the heel-wall junction. Foot angle was measured using a pair of calipers (Modelcraft 5-in-1 Angle Tool and Gauge, Shesto, Watford, UK) at the middle of the dorsal aspect of the claw and adjacent to the sole.

#### Other Explanatory Variables

At each time point, BCS was assessed using a 1-to-5 scale in 0.25 increments (Ferguson et al., 1994). Information regarding animal parity and calving dates were taken from farm records.

### Data Editing

In total, 2,352 cows were enrolled. Data handling is summarized in Supplemental Figure S2 (https://data.mendeley.com/datasets/vk42vz8cht/1; Griffiths, 2023). Two datasets for each time point (T1-precalving and T2-calving) from the initial study population were created. These were initially identical but featured variables relevant to the time point of interest. These datasets were then filtered to remove missing data. Missing data occurred if the animal left the herd, if the animal did not calve during the study period, or if lesion data were incomplete. Animals were also excluded if not sampled at the planned time points within the study design (T1-precalving: −90 to −14 d relative to parturition; T2-calving: 0 to +14 d; T3-early: +40 to +130 d).

This resulted in 1,960 cows for analysis of T1-precalving variables and 2,027 cows for analysis of T2-calving variables with sole lesion as the outcome, and 1,956 cows for analysis of T1-precalving variables and 2,024 cows for analysis of T2-calving variables with white line lesion as the outcome.

The proportion of missing data for each variable was then calculated. All variables were found to be missing <10%; therefore, single imputation based on the mean calculated after grouping by stage, farm, and parity average for each stage and parity-farm group was utilized (Little and Rubin, 2002).

### Variable Processing: Outcome Variables

Due to the significant number of grade 1 (mild) white line and sole hemorrhage lesions observed, in addition to also questions of how important a light pink lesion less than 2 cm in diameter or with diffuse discoloration would be and whether such a lesion could be a reflection of an earlier lesion or very minor damage, we created 2 binary outcome variables: the presence or absence of a sole lesion or white line lesion, both at the T3-early time point. Case animals for sole lesions as an outcome were defined as those with a sole hemorrhage of grade 2 or above or sole ulcer (any grade) on any claw, whereas case animals for white line lesion were required to have a white line lesion of grade 2 or above on any claw. Control animals were required to have all 4 feet checked and the absence of a sole ulcer or a sole hemorrhage (grade 2 or above) for sole lesion and the absence of white line lesions greater than grade 2 on any claw for white line lesions.

### Variable Processing: Explanatory Variables

#### Sole Temperature

Thermography images were processed using FLIR Tools software (version 5.13.18031.2002, FLIR Systems UK, Kent, UK) and the automated maximum temperature search tool. The obtained sole temperature measurements were corrected for ambient temperature as previously described by Anagnostopoulos et al. (2021). Following correction for the ambient temperature, sole temperature for each hind foot was averaged (arithmetic mean) to create a single continuous sole temperature variable at cow level.

#### Digital Cushion Thickness and Sole Horn Thickness

Ultrasonographic images were analyzed using ImageJ software (Schneider et al., 2012) by a single assessor blinded to farm, parity, stage of lactation, and presence of a lesion. Two measurements were taken from the saved images. The first, digital cushion thickness (**DCT**), was measured from the distal phalanx to the interface between the sole horn and the sole soft tissues, and just cranial to the tuberculum flexorum, at the thickest part of the digital cushion. This ultrasonographic measurement of DCT is not exclusively that of the digital cushion, due to the measurement also containing all soft tissues from the distal phalanx to the sole, including connective tissue and the corium (Räber et al., 2004). The term “sole soft tissue thickness” (Newsome et al., 2017a) is, therefore, more accurate; however, for consistency with the majority of published research in this area, and to keep the terms used to describe the sole soft tissue thickness and sole horn thickness more distinct, DCT will be used throughout this article. The second measurement, sole horn thickness (**SHT**), at the same location as DCT, comprised the entire distance from the distal phalanx to the boundary between the sole horn and the standoff; then the DCT was subtracted to calculate the sole horn thickness. Measurements were taken only if recorded landmarks were identifiable with confidence. These identifiable landmarks included the distal phalanx, the tuberculum flexorum, the interface between the sole and the sole soft tissues, and the interface between the sole and the standoff. The DCT and SHT (left hind lateral claw) were treated as continuous variables.

#### Foot Angle and Heel Depth

The average (arithmetic mean) foot angle and heel depth across both hind feet were calculated to create explanatory variables at cow level. Foot angle and heel depth were analyzed as continuous variables. Additionally, to aid interpretation, foot angle was also analyzed as a categorical variable. The foot angle was grouped according to commonly advised recommendations (Archer et al., 2015). The first group consisted of cows with a narrow foot angle (<45°). The second group had a moderate foot angle, in keeping with the current recommendation (45°–52°), and the third group had a wide foot angle (>52°).

#### Other Explanatory Variables

Farm was included as a 4-level categorical variable. Parity was grouped into 3 levels according to their parity at T3-early: primiparous, second lactation, and third lactation or greater. Body condition score was grouped in biologically meaningful groups (≤2.5, 2.75–3.25, ≥3.5); however, due to a small number (<5) of cases for white line models within each group, this was treated as a binary variable (≤3.25 or ≥3.5) for analyses with white line as the outcome. A binary variable to denote the presence of a previous lesion (at either T1-precalving or T2-calving) using the same case definition as the outcome variable was created.

### Statistical Analyses

To test our hypothesis, explanatory variables were analyzed at 2 time points (T1-precalving and T2-calving), against 2 different outcomes (presence of either a sole lesion or white line lesion at T3-early) at the cow level. Data analysis was undertaken using R Studio (R Core Team, 2020), with the Tidyverse (Wickham et al., 2019), Broom (Robinson et al., 2023), performance (Lüdecke et al., 2021), ggeffects (Lüdecke, 2018), and emmeans (Lenth, 2023) packages.

### Univariable Analyses

As part of the exploratory analysis, univariable analyses were undertaken with logistic regression using the glm() function. We also tested the pairwise correlations between explanatory variables (sole temperature, DCT, SHT, foot angle, and heel depth) at each time point.

### Multivariable Analyses

**Figure 1:**
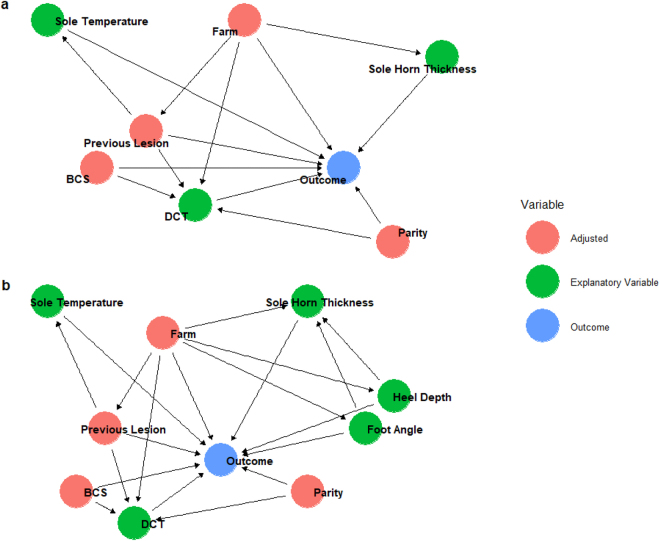
Causal diagrams depicting variables at the T1-precalving (a) and T2-calving time points (b) contributing to the development of the outcome (either sole or white line lesions) at the T3-early lactation time point (Textor et al., 2011; Barrett, 2018). T1-precalving: −90 to −14 d relative to parturition; T2-calving: 0 to +14 d; T3-early: +40 to +130 d. Adjusted = variables identified as having a confounding effect which require conditioning; these were included within the model to limit bias. Explanatory variables = variables of interest within the study; outcome = the presence of a sole lesion or white line lesion depending on the model; DCT = digital cushion thickness.

Multivariable models were built to assess the explanatory variables and the outcome, adjusted for potential and known confounders. Directed acyclic graphs were constructed (Lipsky and Greenland, 2022), with biologically plausible relationships based on prior literature included (Figure 1).

All explanatory variables identified in the directed acyclic graphs were submitted to 4 multivariable fixed effect logistic regression models. The first model examined the relationship between T1-precalving (model 1a) variables on the development of white line lesions. Explanatory and adjusted variables submitted to model 1a were SHT, DCT, sole temperature, farm, BCS, parity, and presence of a white line lesion. The second examined the relationship between T2-calving (model 1b) variables on the development of white line lesions. The explanatory and adjusted variables submitted to model 1b were SHT, DCT, sole temperature, foot angle, heel depth, farm, BCS, parity, and presence of a white line lesion. The third examined the relationship between T1-precalving (model 2a) variables on the development of sole lesions. The explanatory and adjusted variables submitted to model 2a were SHT, DCT, sole temperature, farm, BCS, parity, and presence of a sole lesion. The fourth examined the relationship between T2-calving (model 2b) variables on the development of sole lesions. The explanatory and adjusted variables submitted to the model were SHT, DCT, sole temperature, foot angle, heel depth, farm, BCS, parity, and presence of a sole lesion. Biologically plausible 2-way interactions involving explanatory variables were tested in the model and evaluated by visual inspection, and models were compared with a likelihood ratio test (*P* ≤ 0.05). Models were evaluated using the performance package. Log-linearity was assessed by visualizing scatter plots of explanatory variables against logit values. Multicollinearity was assessed for each explanatory variable by calculating the variance inflation factors (Alin, 2010). Outliers and influential points were evaluated using Cook's distance (Cook, 1977). The Hosmer-Lemeshow test (Lemeshow and Hosmer, 1982) and visualization of binned residuals (Gelman and Hill, 2006) were used to evaluate the model fit, and the coefficient of discrimination (Tjur's R^2^) was calculated to assess the discriminatory power of the model (Tjur, 2009), with 1 corresponding to perfect discrimination between predicted and known disease status and 0 corresponding to no discriminatory power.

As industry recommended levels exist for SHT and foot angle, to aid practical interpretation the model-adjusted probabilities of sole lesion and white line lesion for each category of SHT and foot angle were calculated, averaging for the effects of the other explanatory variables included in the model. Sole horn thickness was categorized as thin (<5 mm), moderate (5–7 mm), or thick (>7 mm; Archer et al., 2015); the arithmetic mean of each level was then determined, and, from this, the adjusted probability was calculated. Foot angle was similarly calculated at 3 levels: the first group consisted of cows with a narrow (<45°) foot angle. The second group had a moderate (45–52°) foot angle, in keeping with the current recommendation, and the third group had a wide (>52°) foot angle (Archer et al., 2015). The arithmetic mean was calculated for each level.

## RESULTS

### Overview of the Dataset

In total, 2,348 cows had measurements recorded at T1-precalving and 2,220 cows at T2-calving. In some cases (n = 38), animals initially enrolled before parturition were removed from any further analysis due to the expected calving not occurring. Reasons for this included abortion, euthanasia due to other health reasons, or cow death. Several records were removed as they were not sampled within the planned study design (Supplemental Figure S2). Most stage records excluded occurred at the T1-precalving time point as the expected calving date was used, based on farm records, which on occasion proved inaccurate. Finally, cows were removed if sole or white line lesion data were missing (Supplemental Figure S2).

Within our study population, the number of case animals for each lesion are presented; 589 (27.8%) and 164 (7.7%) animals displayed a sole lesion or white line lesion respectively. Sole lesion prevalence was highest at T3-early (27.8%), compared with T1-precalving (10.1%) or T2-calving (8.2%). A similar pattern was noted with white line lesion prevalence: lesions were most prevalent at T3-early (7.7%), followed by T1-precalving (6.0%) and T2-calving (5.7%). Descriptive information for explanatory variables is provided in Supplemental Table S2 (https://data.mendeley.com/datasets/vk42vz8cht/1; Griffiths, 2023). At T3-early, the timing of the assessment relative to parturition ranged from 50 to 127 d after calving (mean: 84 d, SD: 13.7).

### Univariable Analyses

Univariable associations between explanatory adjusted variables and the development of a white line lesion during T3-early are detailed in Supplemental Table S3 (https://data.mendeley.com/datasets/vk42vz8cht/1; Griffiths, 2023). Univariable associations between explanatory adjusted variables and the development of a sole lesion during T3-early are detailed in Supplemental Table S4 (https://data.mendeley.com/datasets/vk42vz8cht/1; Griffiths, 2023). Pairwise Pearson correlation coefficients between explanatory variables are described in Supplemental Table S5 (https://data.mendeley.com/datasets/vk42vz8cht/1; Griffiths, 2023).

### Multivariable Analyses: Overview

No violations of the assumptions regarding multicollinearity, log-linearity, and residual distributions were detected. The Hosmer-Lemeshow test statistic was not statistically significant (*P* > 0.05), indicating the fit of each model was acceptable. The model adjusted probabilities of displaying a white line lesion or sole lesion at T3-early were calculated for SHT and foot angle and are detailed in Supplemental Table S6 (https://data.mendeley.com/datasets/vk42vz8cht/1; Griffiths, 2023) or Figure 3.

### Multivariable Analyses: White Line Lesion

#### Model 1a

The first model examined explanatory variables at the T1-precalving time point. The outcome was the presence of a white line lesion at the cow level at T3-early. Model 1a featured 1,956 cows. Tjur's R^2^ value was 0.038. Significance and effect size for explanatory variables are presented in Table 1. A small but statistically significant effect was present for sole temperature, with a 1°C increase associated with an increased odds ratio of 1.04 (95% CI: 1.01–1.07, *P* = 0.009).

**Table 1:** Model 1a: categorical and continuous risk factors at the T1-precalving time point associated with the presence of a white line lesion on any foot at the T3-early lactation time point as the outcome^1^

Variable	Category	Odds ratio	Odds ratio 95% CI	*P*-value
Farm	A	Referent		
	B	0.40	0.21–0.77	0.006
	C	0.38	0.22–0.69	0.001
	D	0.31	0.13–0.72	0.008
Parity	First lactation	Referent		
	Second lactation	1.36	0.72–2.64	0.354
	Third lactation or greater	2.81	1.56–5.26	0.001
Digital cushion thickness (mm)	Continuous variable	1.18	0.97–1.45	0.104
Sole temperature (°C)	Continuous variable	1.04	1.01–1.07	0.009
Sole horn thickness (mm)	Continuous variable	1.04	0.96–1.13	0.342
Presence of a white line lesion at T1-precalving	No white line lesion	Referent		
	White line lesion present	1.96	1.09–3.35	0.018
BCS	≤3.25	Referent		
	≥3.5	1.18	0.80–1.73	0.405

1 The model featured 146 (7.5%) case animals and 1,810 (92.5%) control animals. Odds ratios for continuous variables are calculated for every 1-unit increase. T1-precalving: −90 to −14 d relative to parturition.

#### Model 1b

The second model examined explanatory variables at the T2-calving time point. The outcome of the model was presence of a white line lesion at the cow level at T3-early. Model 1b featured 2,024 cows, and the significance and effect size for explanatory variables can be seen in Table 2. Tjur's R^2^ value was 0.091. A 1°C increase in sole temperature was associated with a very small reduction in the odds of developing a white line lesion (odds ratio [**OR**]:0.96, CI: 0.93–0.99, *P* = 0.006).

**Table 2:** Model 1b: categorical and continuous risk factors at the T2-calving time point associated with the presence of a white line lesion on any foot at the T3-early lactation time point as the outcome^1^

Variable	Category	Odds ratio	Odds ratio 95% CI	*P*-value
Farm	A	Referent		
	B	0.52	0.27–1.03	0.056
	C	0.44	0.24–0.84	0.010
	D	0.25	0.10–0.58	0.002
Parity	First lactation	Referent		
	Second lactation	1.25	0.68–2.35	0.472
	Third lactation or greater	2.68	1.54–4.85	0.001
Digital cushion thickness (mm)	Continuous variable	1.11	0.90–1.37	0.330
Sole temperature (°C)	Continuous variable	0.96	0.93–0.99	0.006
Sole horn thickness (mm)	Continuous variable	1.03	0.95–1.12	0.409
Foot angle (°)	Continuous variable	0.95	0.90–1.01	0.129
Heel depth (mm)	Continuous variable	1.00	0.96–1.05	0.858
Presence of a white line lesion at T2-calving	No white line lesion	Referent		
	White line lesion present	5.49	3.47–8.58	<0.001
BCS	≤3.25	Referent		
	≥3.5	0.90	0.63–1.29	0.572

1 The model featured 155 (7.7%) case animals and 1,869 (92.3%) control animals. Odds ratios for continuous variables are calculated for every 1-unit increase. T2-calving: 0 to +14 d; T3-early: +40 to +130 d.

### Multivariable Analyses: Sole Lesion

#### Model 2a

**Figure 2:**
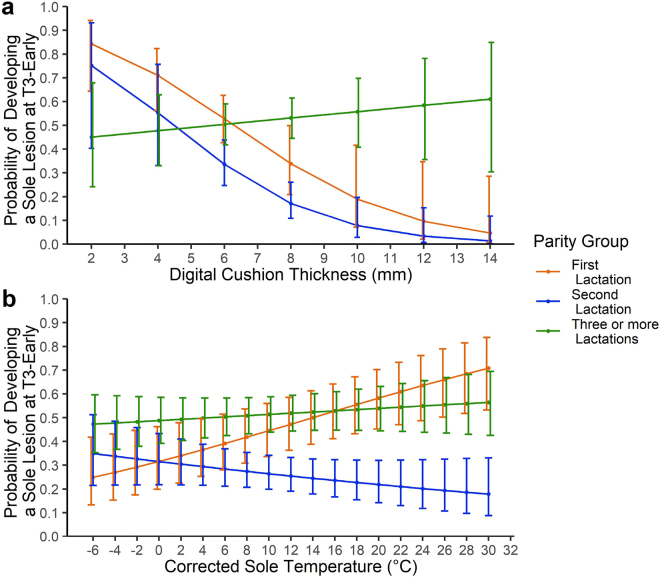
Interactions presented from the fixed effect logistic regression model (model 2a) examining the association between foot conformation traits at the T1-precalving time point and the development of sole lesions during early lactation (T3-early). (a) The interaction between digital cushion thickness (mm) and parity on the model-predicted probability of developing a sole lesion at T3-early. (b) The interaction between ambient corrected sole temperature and parity on the model-predicted probability of developing a sole lesion at T3-early. Error bars describe confidence intervals. T1-precalving: −90 to −14 d relative to parturition; T2-calving: 0 to +14 d; T3-early: +40 to +130 d.

**Figure 3:**
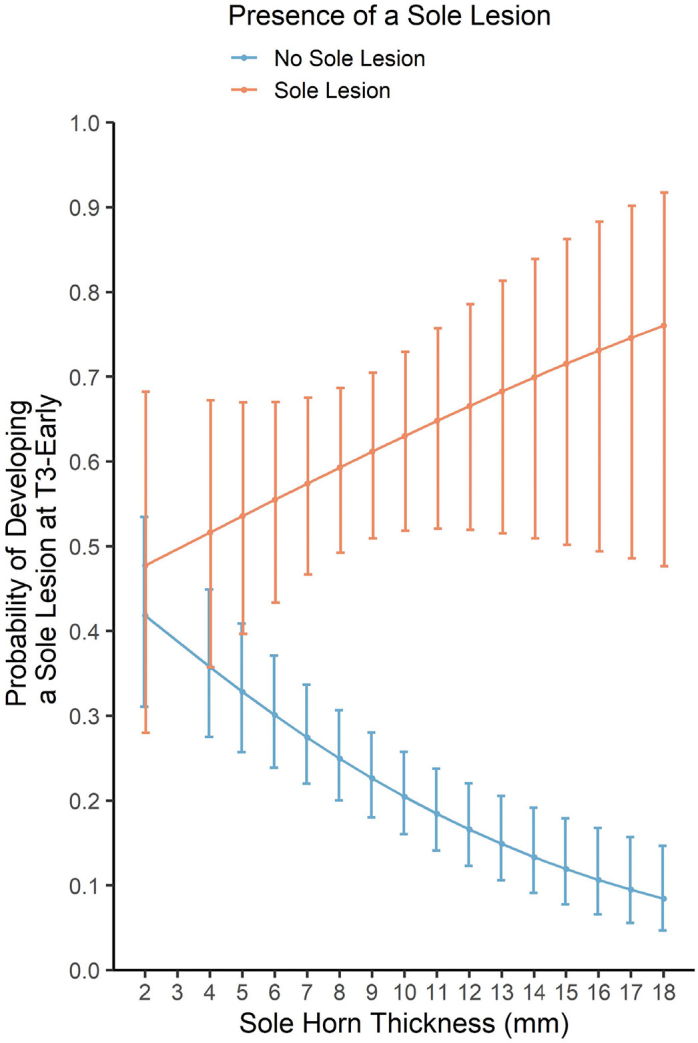
Interaction presented from the fixed effect logistic regression model (model 2a) examining the association between foot conformation traits at the T1-precalving time point and the development of sole lesions during early lactation (T3-early): the interaction between the presence of a sole lesion at T1-precalving and sole horn thickness on the model-predicted probability of developing a sole lesion at T3-early. Error bars describe confidence intervals. T1-precalving: −90 to −14 d relative to parturition; T2-calving: 0 to +14 d; T3-early: +40 to +130 d.

The first model examined explanatory variables at the T1-precalving time point. The outcome of the model was the presence of a sole lesion at the cow level at T3-early. Model 2a featured 1,960 cows. The Tjur R^2^ was 0.125. Significance and effect sizes are presented in Table 3. Digital cushion thickness was significantly associated with the development of sole lesions (OR: 0.68, 95% CI: 0.52–0.87, *P* = 0.003); however, an interaction occurred between DCT and parity (second lactation OR: 0.95, 95% CI: 0.63–1.41, *P* = 0.787; third or greater lactation OR: 1.56, 95% CI: 1.14–2.15, *P* = 0.006; Figure 2a). Sole temperature was also significantly associated with the development of sole lesions (OR: 1.06, 95% CI: 1.02–1.09, *P* = 0.002); however, an interaction with parity was detectable (second lactation OR: 0.92, 95% CI: 0.88–0.97, *P* = 0.001; third or greater OR: 0.96, 95% CI: 0.92–1.00, *P* = 0.033; Figure 2b). Finally, SHT was significantly associated (OR: 0.88, 95% CI: 0.83–0.93, *P* < 0.001) with the development of sole lesions; however, there was an interaction with whether a sole lesion was already present (sole lesion present, OR: 1.23, 95% CI:1.09–1.40, *P* = 0.001; Figure 3).

**Table 3:** Model 2a: categorical and continuous risk factors at the T1-precalving time point associated with the presence of a sole lesion on any foot at the T3-early lactation time point as the outcome^1^

Variable	Category	Odds ratio	Odds ratio 95% CI	*P*-value
Farm	A	Referent		
	B	0.44	0.27–0.73	0.002
	C	0.66	0.41–1.05	0.077
	D	1.02	0.58–1.79	0.956
Parity	First lactation	Referent		
	Second lactation	1.45	0.10–20.93	0.782
	Third lactation or greater	0.10	0.01–0.80	0.031
Digital cushion thickness (mm)	Continuous variable	0.68	0.52–0.87	0.003
Sole horn thickness (mm)	Continuous variable	0.88	0.83–0.93	<0.001
Sole temperature (°C)	Continuous variable	1.06	1.02–1.09	0.002
Presence of a sole lesion at T1-precalving	No	Referent		
	Yes	0.84	0.27–2.59	0.764
BCS	≤2.5	0.84	0.40–1.70	0.640
	2.75–3.25	Referent		
	≥3.5	0.75	0.57–0.98	0.036
Parity × digital cushion thickness (mm)	First lactation × 1-mm increase	Referent		
	Second lactation × 1-mm increase	0.95	0.63–1.41	0.787
	Third lactation or greater × 1-mm increase	1.56	1.14–2.15	0.006
Parity × sole temperature (°C)	First lactation × 1°C increase	Referent		
	Second lactation × 1°C increase	0.92	0.88–0.97	0.001
	Third lactation or greater × 1°C increase	0.96	0.92–1.00	0.033
Sole horn thickness (mm) × presence of a sole lesion at T1-precalving	No lesion present	Referent		
	Lesion present and 1-mm increase in sole horn thickness	1.23	1.09–1.40	0.001

1 The model featured 524 (26.7%) case animals and 1,436 (73.3%) control animals. Odds ratios for continuous variables are calculated for every 1-unit increase. T1-precalving: −90 to −14 d relative to parturition; T3-early: +40 to +130 d.

#### Model 2b

The second model examined explanatory variables at the T2-calving time point. The outcome of this model was the presence of a sole lesion at the cow level at T3-early. The Tjur R^2^ was 0.136. Model 2b featured 2,027 cows. Significance and effect sizes are presented in Table 4. Digital cushion thickness (OR: 0.74, 95% CI: 0.65–0.84, *P* < 0.001), SHT (OR: 0.88, 95% CI: 0.83–0.93, *P* < 0.001), and sole temperature (OR: 1.03, 95% CI: 1.02–1.05, *P* < 0.001) were all associated with the development of sole lesions.

**Table 4:** Model 2b: categorical and continuous risk factors at the T2-calving time point associated with the presence of a sole lesion on any foot at the early lactation time point (T3-early) as the outcome^1^

Variable	Category	Odds ratio	Odds ratio 95% CI	*P*-value
Farm	A	Referent		
	B	0.40	0.25–0.65	<0.001
	C	0.51	0.32–0.79	0.003
	D	0.95	0.56 –1.60	0.845
Parity	First lactation	Referent		
	Second lactation	0.36	0.26–0.50	<0.001
	Third lactation or greater	1.27	0.94–1.73	0.123
Digital cushion thickness (mm)	Continuous variable	0.74	0.65–0.84	<0.001
Sole horn thickness (mm)	Continuous variable	0.88	0.83–0.93	<0.001
Sole temperature (°C)	Continuous variable	1.03	1.02–1.05	<0.001
Foot angle	Continuous variable	1.00	0.97–1.05	0.821
Heel depth	Continuous variable	1.00	0.97–1.02	0.748
Presence of a sole lesion at T2-calving	No	Referent		
	Yes	3.37	2.34–4.86	<0.001
BCS	≤2.5	2.86	1.50–5.47	0.001
	2.75–3.25	Referent		
	≥3.5	0.99	0.79–1.25	0.943

1 The model featured 554 (27.3%) case animals and 1,473 (72.7%) control animals. Odds ratios for continuous variables are calculated for every 1-unit increase. T2-calving: 0 to +14 d; T3-early: +40 to +130 d.

## DISCUSSION

An increased SHT at T1-precalving or T2-calving was associated with reduced odds of developing a sole lesion. The reported normal SHT is 8 to 15 mm at the heel-sole junction (van Amstel et al., 2002), and the average SHT in our study was within the reported normal range at both time points. Some variation was noted, with 67 out of 1,982 (3.38%) cows before calving and 23 out of 2,082 (1.1%) cows immediately after calving displaying thin soles when using the recommended minimum SHT of 5 mm (Archer et al., 2015). Thin soles result from overtrimming or an imbalance between horn growth and horn wear. Increased wear can be triggered by housing factors or by increased water content in the claw horn (Manske, 2002; van Amstel et al., 2002; Führer et al., 2019). Most weight bearing is shared between the sole, the heel bulb, and the wall of the claw (van der Tol et al., 2003). The weight-bearing capacity of the wall is reduced as the sole comes into increasing contact, due to increased wear (Telezhenko et al., 2008). A thin sole is thought to disrupt load bearing, triggering compression of the corium and damage to keratinocytes, resulting in sole hemorrhage and sole ulcers (Archer et al., 2015).

The presence or absence of a sole lesion before calving significantly affected whether a thicker sole was protective or whether it increased the risk of that animal developing a lesion during early lactation. We found that animals with both a sole lesion before calving and a thicker sole were at greater risk of developing a subsequent lesion during early lactation. Lameness has been shown to change animal behavior, with lame cows spending more time resting (Blackie et al., 2011; Weigele et al., 2018), less time moving around their housing (Singh et al., 1993), and less time in feeding areas (Palmer et al., 2012; Vázquez Diosdado et al., 2018). This change in behavior before calving alters weight bearing, resulting in thicker soles due to reduced horn wear and subsequent higher local maximum pressures in softer parts of the claw such as the heel and the sole (van der Tol et al., 2003; van der Tol et al., 2004). Animals that have a history of lameness-causing lesions are predisposed to suffer subsequent lesions, with exostosis on the palmar or plantar aspect of the distal phalanx hypothesized to be an exacerbating factor (Foditsch et al., 2016; Newsome et al., 2016; Randall et al., 2016). Exostosis compromises the claw's functional capacity to cope with the local pressure applied to the sole during movement and therefore may compound the threat from overloading, possibly at a thickness of sole previously considered normal. Altered weight bearing onto non-painful claws in combination with possible exostosis from previous lesions could also increase the risk of subsequently developing a sole lesion.

A thin digital cushion before and shortly after calving was associated with increased odds of developing a sole lesion during the early lactation period. The digital cushion protects the sensitive horn-producing keratinocytes within the corium during normal weight bearing and ambulation. Our results are consistent with several studies associating a thin digital cushion in dairy cows with CHDL (Bicalho et al., 2009; Machado et al., 2011). Our findings are in agreement with Newsome et al. (2017a), Stambuk et al. (2019), and Griffiths et al. (2020), who described cows with thin digital cushions to have an increased risk of subsequent lameness and lesion occurrence. Two main mechanisms explain the association between thin digital cushions and the development of CHDL. The first is that of tissue mobilization. The digital cushion is composed of fat and connective tissue (Räber et al., 2004). Cows are at increased risk of fat mobilization during early lactation to support the demands of milk production. Griffiths et al. (2020) found that a one-point increase in BCS was associated with a 0.3-mm estimated increase in the digital cushion depth, and although Newsome et al. (2017b) found no such association with BCS, a 10-mm difference in measured back fat thickness was associated with a 0.13-mm difference in DCT. Rather than the digital cushion becoming depleted, it is alternatively hypothesized that these tissues become compressed through a weakening of the suspensory apparatus, resulting in sinkage of the distal phalanx. A significant association between parturition and laxity of the suspensory apparatus has been found. This “calving effect” has been noted to be fibrogenic in origin and hypothesized to be the result of hormonal changes associated with normal parturition (Knott et al., 2007).

Although overall a thick digital cushion before calving is associated with reduced odds of developing a sole lesion, animals in their third or greater lactation with thicker digital cushions had increased odds compared with their lower-parity herd mates. First-lactation animals display sparse amounts of fat, with the digital cushion mainly composed of loose connective tissue instead. As parity increases, fat becomes the dominant material. Beyond the third parity, however, the digital cushion overall is reduced in size and the composition is dominated by connective tissue (Räber et al., 2004). A history of CHDL is associated with chronic damage within the foot, which increases the likelihood of cows with a history of lameness developing further lesions (Newsome et al., 2016; Randall et al., 2016; Wilson et al., 2021). Damage acquired from previous lameness events could be responsible for the overall reduction in size, as chronic inflammation leads to a greater proportion of connective tissue and mineralized tissue deposition. Due to the compromised function of the DCT, these animals would be at an increased risk of acute inflammation associated with a lameness event shortly before enrolment, which may result in an accompanying increase in the thickness of the digital cushion. Although DCT was associated with sole lesions, it was not associated with white line lesions before or after calving. Given that the digital cushion is proposed to support the tissues underlying the pedal bone, the lack of association with white line lesions is perhaps not unexpected. Newsome et al. (2017a) also found a lack of association, although this was likely due to a small number of white line lesions within their cohort.

In our study, an increase of 1°C in sole temperature shortly after calving was associated with the development of a sole lesion during the early lactation period. Previous studies have found association between an increased foot temperature at the time of diagnosis of sole lesions (Main et al., 2012), foot lesions (Stokes et al., 2012), or lameness (Oikonomou et al., 2014; Rodríguez et al., 2016). However, Wilhelm et al. (2015) did not find a significant association between a total claw lesion score and concurrent claw temperature. Although our study found a small effect size, lesions found on feet have typically recorded increased temperatures of between 2.5°C and 5°C (Main et al., 2012; Stokes et al., 2012). Sole horn has been highlighted as a poor conductor of heat (Jantscher et al., 2003); therefore the increase in temperature associated with inflammation could be underestimated. If thermography is a proxy measure for the inflammatory status of the corium, then increased sole temperatures shortly after calving could be indicative that the corium is inflamed at this time. Parturition results in substantial biomechanical changes within the bovine hoof, including increased laxity of the suspensory apparatus (Tarlton et al., 2002; Knott et al., 2007). Although this “calving effect” on the hoof connective tissue is a fibrogenic change (Tarlton et al., 2002), these changes could lead to an inflammatory response should the corium become contused, with sole lesions becoming visible as the horn above wears away. This is somewhat supported by Wilson et al. (2022): when cows were treated with a nonsteroidal anti-inflammatory drug at calving and at treatment for lameness, in addition to prompt and effective treatment of lameness events during the study, the authors found a significantly reduced probability of lameness compared with cows only receiving a block when trimmed.

An increased foot angle at T2-calving was not significantly associated with either sole lesion or white line lesion development in the multivariable analysis. Foot angle is included in the lameness advantage index, which is associated with the incidence of sole hemorrhage, sole ulcers, and lameness (Barden et al., 2022). van Dorp et al. (2004) highlighted that cows with a steeper foot angle trait had genetically significantly better locomotion, while Kougioumtzis et al. (2014) found that an intermediate foot angle breeding trait was related to reduced lameness, with a steeper angle preferable. A shallow foot angle trait was associated with worse locomotion (Boelling and Pollott, 1998). Cows had an 8% reduction in the odds of being clinically lame (based on mobility scores) when the foot angle increased by 1° (Wells et al., 1993). However, we measured foot angle before the lesion of interest occurred, whereas the Wells et al. (1993) study measured clinical lameness at the same time that foot angle was recorded.

Heel depth was not significantly associated with the development of either sole or white line lesion when other foot anatomy variables are considered. Heel depth has been suggested to be the least useful trait to assess the shape and size of dairy cattle feet (Boelling and Pollott, 1998).

Sole horn thickness, foot angle, and heel depth are all key characteristics used by foot trimmers when undertaking preventative foot trimming. Our results suggest that SHT is the most important of these characteristics. Sole horn thickness was significantly associated with the development of sole lesions in the multivariable analysis when all other foot anatomy traits were accounted for, whereas foot angle and heel depth were not significantly associated with lesion development. Sole horn thickness is therefore likely the variable of most importance in the development of sole lesions. Our study did not feature a large number of cows with extreme foot angles or cows with overgrown sole horn (Supplemental Table S2), and therefore the conclusions drawn should reflect normal preventative trims. The role of foot angle in the development of CHDL may therefore be more important in herds with poor routine foot trimming, resulting in extreme foot angles. Overtrimming the individual cow is an important consideration, and enough sole horn should be left in situ that the sole horn can provide the claws with adequate protection from the environment. Sole horn thickness measurements collected via ultrasonography showed high correlation coefficients (Kofler et al., 1999; Laven et al., 2012) between ultrasonographic and anatomical measurements. A study by Bach et al. (2019) demonstrated a correlation coefficient of 0.7; however, those authors highlighted that thicker soles were likely to be underestimated using ultrasonography. Our study showed that cows with thicker soles were less likely to develop sole lesions. The current recommendation of 5 to 7 mm (Davy et al., 2023) may need to be reconsidered in favor of a thicker sole. Given that our results show that thicker sole horn is protective even when likely underestimated, a conservative recommendation of 10 mm may be more appropriate, but further research to test this hypothesis is needed.

There are limitations to our study, which need to be taken into consideration when interpreting our findings. Our results are from cows enrolled from only 4 dairy farms, which, despite featuring operating practices common to many UK commercial dairy farms, could not be considered representative of the full spectrum of dairy farms. At T3-early, DIM varied; however, as sole lesions were grouped into severe sole hemorrhage and sole ulcers, the authors do not anticipate that this variation will affect the case definition of these animals. Furthermore Newsome et al. (2017b) found DCT to be very similar across 2 time points, which roughly correspond to the range of DIM featured in our study. Although more than 90% of lesion assessments were undertaken by a single person, no formal measure of agreement between researchers was calculated. A small risk exists that, when only a small amount of horn was removed to detect lesions in primiparous animals at T1-precalving and T2-calving, small deep lesions may have been missed. This may have affected whether a previous lesion was noted during T1-precalving or T2-calving for heifers. The wider modeling was still apparent in multiparous animals at T2-calving, and therefore the removal of a thin layer of horn would have revealed the presence of any lesions. This study used detailed lesion records collected during the study period; we did not, however, include lesion history before enrolment in the study, due to the variable reporting quality of lesion data by farms. As previously discussed, SHT was measured using stored ultrasound images; cows with a thicker sole were likely to be underestimated using ultrasonography (Bach et al., 2019). Measurement of SHT is not easy to accomplish; pressure applied to the sole has been advocated but is subjective (van Amstel et al., 2003). We measured sole temperature at the sole surface, and the size of the sole horn may affect how much heat is emitted from the foot, thereby affecting the heat signature recorded. Gross contamination of the foot was removed using a paper towel, sole temperature was corrected for ambient temperature, and the foot was not trimmed before measurement; therefore several of the key factors previously associated with changes to foot temperature were appropriately managed. The assessor was blinded to lesion presence and stage but not farm when reading the thermography images; however, the software tool used is automated, and therefore the risk of bias is low. We analyzed our data at the cow level, and the association between the presence of a previous sole or white line lesion and a subsequent lesion during early lactation is therefore not claw specific. Newsome et al. (2017a) reported results similar to ours when analyzing claw-level data. No significant difference in digital cushion volume has been found between the hind limbs (Wilson et al., 2021); therefore we believe that the hind left lateral claw is an adequate proxy for that of the hind right lateral claw, given that most lesions occur in the lateral claw of the hind feet (Murray et al., 1996). No conclusion can be drawn about the relative content of connective tissue to adipose tissue in the DCT, due to the method of imaging used, the composition of which may affect the capacity of force dissipation. Finally, the ultrasound measurements recorded were on lifted feet. Bach et al. (2019) have developed a novel method of scanning the digital cushions while weight bearing, and although the sample size is small, it does suggest that the weight bearing yields different DCT measurements to lifted feet.

There are, however, several strengths to our study: it is the largest study of its kind, and a large number of detailed lesion records were collected primarily by a single experienced assessor. The explanatory variables were measured objectively using ultrasound, infrared thermography, and calipers, and these thermographic and ultrasound images were both assessed after data collection ceased by a single assessor blinded to parity, lesion, and stage and, in the case of the ultrasonographic assessor, to farm.

## CONCLUSIONS

The results from our prospective cohort study confirm the importance of DCT in the occurrence of sole lesions during the early lactation period. We suggest a potential disparity in etiopathogenesis between sole lesions and white line lesions. Foot angle and heel depth at calving were not found to be significantly associated with the development of either sole lesions or white line lesions; however, SHT was shown in our study to be significantly associated with the risk of developing sole lesions, and the direction of this association before calving was dependent on the presence of a sole lesion at the time of measurement. An increased sole temperature was associated with the development of both sole and white line lesions. Particular care should be taken when trimming and managing cows so that adequate SHT is maintained.
